# Regulation of Serum Amyloid A3 (SAA3) in Mouse Colonic Epithelium and Adipose Tissue by the Intestinal Microbiota

**DOI:** 10.1371/journal.pone.0005842

**Published:** 2009-06-09

**Authors:** Christopher S. Reigstad, Gunnel Östergren Lundén, Jenny Felin, Fredrik Bäckhed

**Affiliations:** 1 Sahlgrenska Center for Cardiovascular and Metabolic Research/Wallenberg Laboratory, University of Gothenburg, Gothenburg, Sweden; 2 Department of Molecular and Clinical Medicine, University of Gothenburg, Gothenburg, Sweden; Columbia University, United States of America

## Abstract

The gut microbiota has been proposed as an environmental factor that affects the development of metabolic and inflammatory diseases in mammals. Recent reports indicate that gut bacteria-derived lipopolysaccharide (LPS) can initiate obesity and insulin resistance in mice; however, the molecular interactions responsible for microbial regulation of host metabolism and mediators of inflammation have not been studied in detail. Hepatic serum amyloid A (SAA) proteins are markers and proposed mediators of inflammation that exhibit increased levels in serum of insulin-resistant mice. Adipose tissue-derived SAA3 displays monocyte chemotactic activity and may play a role in metabolic inflammation associated with obesity and insulin resistance. To investigate a potential mechanistic link between the intestinal microbiota and induction of proinflammatory host factors, we performed molecular analyses of germ-free, conventionally raised and genetically modified *Myd88*−/− mouse models. SAA3 expression was determined to be significantly augmented in adipose (9.9±1.9-fold; *P*<0.001) and colonic tissue (7.0±2.3-fold; *P*<0.05) by the presence of intestinal microbes. In the colon, we provided evidence that SAA3 is partially regulated through the Toll-like receptor (TLR)/MyD88/NF-kappaB signaling axis. We identified epithelial cells and macrophages as cellular sources of SAA3 in the colon and found that colonic epithelial expression of SAA3 may be part of an NF-kappaB-dependent response to LPS from gut bacteria. *In vitro* experiments showed that LPS treatments of both epithelial cells and macrophages induced SAA3 expression (27.1±2.5-fold vs. 1.6±0.1-fold, respectively). Our data suggest that LPS, and potentially other products of the indigenous gut microbiota, might elevate cytokine expression in tissues and thus exacerbate chronic low-grade inflammation observed in obesity.

## Introduction

Genetic variations influence individual susceptibility to obesity and insulin resistance, however, lifestyle and environmental factors play key roles in the development of these diseases [1]. The gut microbiota has recently been suggested as one such environmental factor [2], [3]. Accordingly, obesity is associated with an altered gut microbiota in both mice and humans [4], [5], [6] and decreased microbial diversity [7]. Moreover, evidence from studies of germ-free (GF) mice indicates that the gut microbiota may promote obesity by increasing nutrient absorption from the gut and reducing peripheral fatty acid oxidation [8], [9].

Obesity and the metabolic syndrome are associated with low-grade ‘metabolic’ inflammation [10] and recent data suggest that lipopolysaccharide (LPS) derived from the gut microbiota contributes to the increased development of adipose tissue and impaired glucose tolerance [11], [12]. LPS signaling is mediated through the Toll-like receptor 4 (TLR4)/MyD88/NF-κB signaling pathway [13]. Mice deficient in TLR4 or its co-receptor CD14 are protected against diet-induced obesity and insulin resistance [14], [15]. However, it is not clear which inflammatory molecules are induced by LPS or other products of the gut microbiota.

Serum amyloid A (SAA) proteins are proposed mediators of inflammation and metabolism, with increased serum levels being associated with obesity, chronic hyperglycemia, insulin resistance and cardiovascular disease [16], [17], [18], [19], [20], [21]. Thus, SAA may be one of the potential factors linking chronic inflammation and the development of obesity. In mice, four functional SAA isoforms have been identified: SAA1-4 [22]. SAA3 is primarily expressed extrahepatically and is the most abundant SAA isoform in both adipose tissue [18], [23] and the mouse colon [24]. A recent study showed that SAA3 is upregulated in the adipose tissue of mice fed a high-fat diet, and the authors proposed that SAA3 could be a mediator of the chronic inflammation associated with insulin resistance in obesity [18]. Elevated expression of SAA3 is also observed in the intra-abdominal fat of genetically obese (*ob*/*ob*) mice compared with that of wild type controls [25].

Intraperitoneal administration of LPS induces SAA3 expression in adipose tissue *in vivo* [26]; however, little is known about the inducibility of SAA3 by LPS in other tissues expressing SAA3. This prompted us to compare expression of SAA3 mRNA in adipose and intestinal tissue from GF and conventionally raised (CONV-R) mice to determine whether components of the gut microbiota could augment SAA3 levels in these tissues. We investigated a potential signaling pathway responsible for microbiota-dependent increases in SAA3 expression in mice, as well as macrophage and epithelial cell culture models.

## Results

### Gut Microbiota Augments Adipose SAA3 mRNA Expression

**Figure 1:**
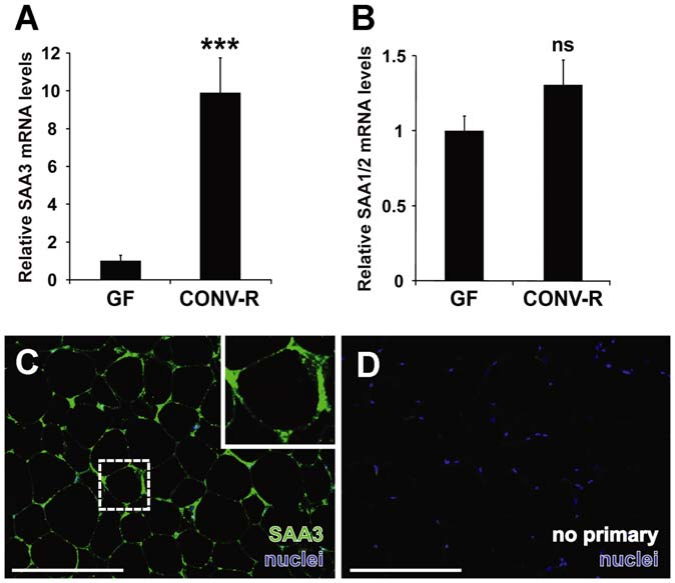
SAA3 but not SAA1/2 mRNA expression is increased in mouse adipose tissue in the presence of microbiota. (A) qRT-PCR analysis of SAA3 mRNA expression levels in epididymal adipose tissue of germ-free (GF) and conventionally raised (CONV-R) mice (n = 10 mice per group). (B) qRT-PCR analysis of SAA1/2 mRNA expression levels in epididymal adipose tissue of germ-free (GF) and conventionally raised (CONV-R) mice (n = 10 mice per group). Data are means±SEM. ***P<0.001; Student's *t*-test. (C) Representative immunostaining of SAA3 (*green*) and *bis*-benzimide-labeled nuclei (*blue*) in CONV-R mouse epidiymal adipose tissue. (D) Primary antibody omitted. Scale bars = 200 µm.

The microbiota induces adipocyte hypertrophy and weight gain in mice and acts as an environmental modulator of fat storage [8]. To address whether expression of SAA isoforms in adipose tissue could be altered by the microbiota, we assessed SAA1/2 and SAA3 mRNAs by quantitative real-time PCR (qRT-PCR) in GF and CONV-R Swiss-Webster mice (n = 10 per group). mRNA levels of SAA3 in adipose tissue were significantly higher (9.9-fold) in CONV-R mice compared with GF mice (Figure 1A). In contrast, adipose tissue from CONV-R mice did not exhibit significantly increased SAA1/2 mRNA expression compared with GF mice (Figure 1B).

Immunohistochemical analysis of SAA3 protein revealed intense staining of epididymal adipose tissue (Figure 1C) with minimal background in treatments omitting the primary SAA3 antibody (Figure 1D). The staining pattern suggest that adipocytes are positive for SAA3, consistent with a previous report [17]; however, interstitial macrophages may also contribute to SAA3 expression in adipose tissue [8]. Similar spatial localization patterns were identified in mesenteric and perinephric adipocytes (Figure S1).

### Gut Microbiota Augments SAA3 mRNA Expression in the Colonic Epithelium

**Figure 2:**
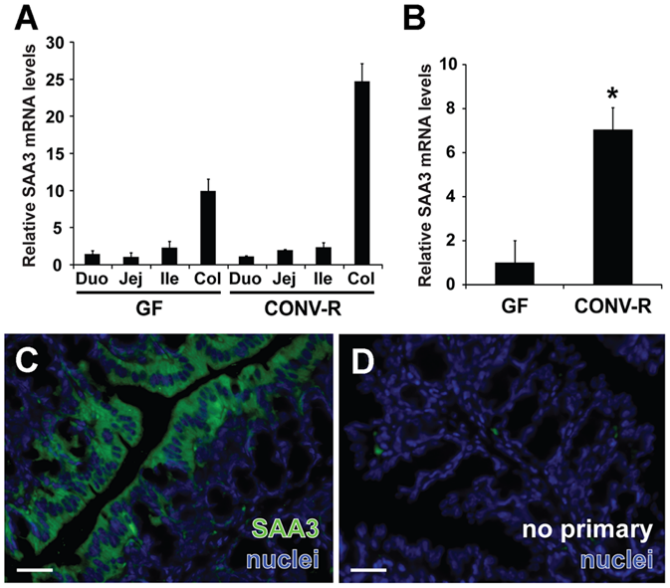
SAA3 mRNA expression is increased in mouse colon in presence of microbiota. (A) qRT-PCR analysis of SAA3 mRNA expression levels along the length of the gut in germ-free (GF) and conventionally raised (CONV-R) mice using pooled cDNA samples (n = 5 mice per group). *Duo*, duodenum; *Jej*, jejunum; *Ile*, ileum; *Col*, proximal colon. Data are means±SD. (B) Colonic SAA3 mRNA expression assayed in individual mice (n = 8−10 mice per group). Data are means±SEM. *P<0.05; Student's *t*-test. (C) Representative immunohistochemical staining of CONV-R mouse colonic sections shows *bis*-benzimide-positive nuclei (*blue*) and SAA3 (*green*) expression in surface epithelial cells. (D) Primary antibody omitted. Scale bars = 30 µm.

The role of the microbiota in the expression of SAA3 along the gut is unknown. To investigate the spatial distribution of SAA3, we assessed relative mRNA expression in pooled cDNAs from duodenum, jejunum, ileum and proximal colon (n = 5 mice per group). We found that the expression of SAA3 mRNA was highest in the colon in both GF and CONV-R mice (Figure 2A). To assess whether the gut microbiota significantly altered colonic SAA3 expression, we analyzed a larger group of individual mice (n = 8−10 per group) and showed that colonic SAA3 mRNA levels were significantly higher in CONV-R mice compared with GF mice (7.0-fold; Figure 2B). Immunohistochemical analysis of SAA3 expression in the colon revealed strong staining in the mucosal layer (Figures 2C–2D).

### Gut Microbiota Augments TNF-α mRNA Expression in the Colon

**Figure 3:**
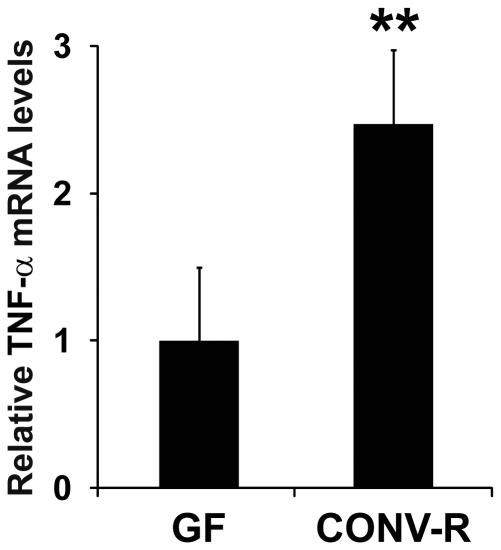
TNF-α mRNA is increased in the mouse colon in the presence of microbiota. qRT-PCR analysis of SAA3 mRNA expression levels in colonic tissue of germ-free (GF) and conventionally raised (CONV-R) mice (n = 10 mice per group). Data are means±SEM. **P<0.01; Student's *t*-test.

TNF-α is one of the known regulators of SAA3 expression and a widely expressed cytokine with potential roles in chronic inflammatory disease [17], [27]. As revealed by qRT-PCR, TNF-α mRNA expression was significantly increased in the colons of CONV-R mice compared with GF mice (2.5-fold; Figure 3).

### Augmentation of Colonic SAA3 Expression by TLR4/MyD88/NF-κB Signaling

**Figure 4:**
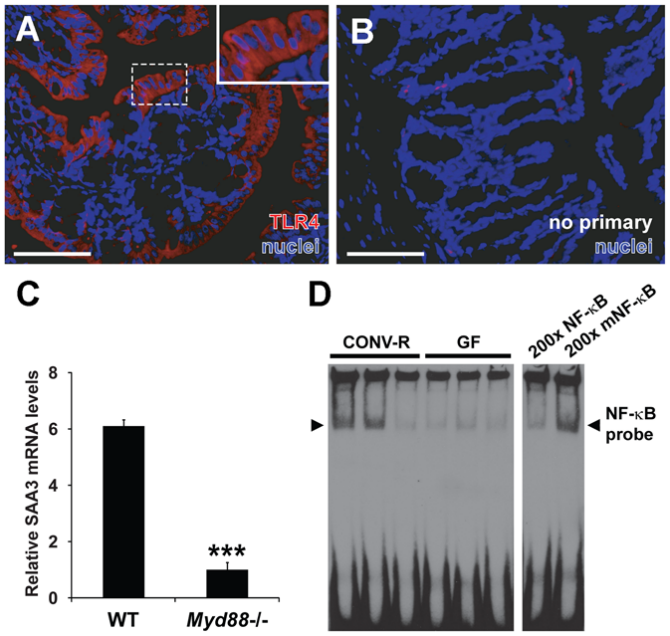
SAA3 expression in the mouse colon may be stimulated by TLR/MyD88/NF-κB signaling. (A) Representative immunostaining for TLR4 (*red*) and *bis*-benzimide-positive nuclei (*blue*) in CONV-R mice. (B) Primary antibody omitted. Scale bars = 100 µm. (C) qRT-PCR analysis of colonic SAA3 mRNA expression levels in microbiota-associated wild-type (WT) and *Myd88−/−* mice (n = 10−13 mice per group). ***P<0.001; Student's *t*-test. (D) Representative electrophoretic mobility shift assay depicting binding of nuclear proteins to biotinylated probes containing an NF-κB binding site in three conventionally raised (CONV-R) (lanes 1–3) and three germ-free (GF) mice (lanes 4–6). The two right-hand lanes show addition of a 200-fold excess of unlabeled normal (200× NF-κB) or mutated (200× mNF-κB) probe in competition with the biotinylated NF-κB probe.

We next investigated whether microbe-dependent modulation of colonic SAA3 expression could be mediated through the TLR4/MyD88/NF-κB signaling axis. TLR4, the signaling receptor for bacterial LPS, has previously been shown to be expressed by intestinal epithelial cell lines and ileal enterocytes *in vivo* [28], [29]. Here, we verified that TLR4 could be detected in the surface epithelial cells of the mouse colon (Figures 4A–4B). TLR4 staining of the colonic epithelium was observed in all experimental groups used in this study, with no significant differences in relative colonic TLR4 mRNA levels between GF and CONV-R mice (data not shown).

MyD88 is an adaptor protein required for downstream signaling of most TLRs in mice and humans [30]. To test whether MyD88 signaling is required for increased colonic SAA3 expression, we compared SAA3 mRNA levels in the intestines of WT C57Bl/6 mice and isogenic *Myd88*−/− mutants; both of these groups were conventionally raised (*i.e.*, in the presence of intestinal microbiota) but maintained on a high-fat, high-sugar diet for eight weeks to promote SAA3 expression [17]. Quantification of colonic SAA3 mRNA in individual mice (n = 10−13 mice per group) revealed decreased SAA3 in *Myd88*−/− mice compared with WT (Figure 4C). The levels of SAA3 mRNA in epididymal fat from these *Myd88*−/− mice were also significantly lower than those of their WT counterparts (Figure S2).

Downstream of TLR4 and MyD88, activated NF-κB can bind the SAA3 promoter [31] and has been shown to regulate SAA3 transcription in hepatoma-derived cell lines and mouse adipocytes [23], [31], [32]. To investigate whether CONV-R mice exhibited increased DNA binding of NF-κB, we performed EMSAs of nuclear protein extracts from colons obtained from CONV-R and GF mice. We detected NF-κB binding in a total of five out of six CONV-R mice but not in their GF counterparts. A representative EMSA depicting results from three mice per group is shown in Figure 4D. Elevated probe binding in these EMSAs of CONV-R compared with GF colon could be caused by increased NF-κB nuclear translocation or increased NF-κB expression. Together, these findings suggest that the TLR/MyD88/NF-κB signaling axis could partially mediate increased SAA3 expression in the distal gut in the presence of gut microbiota.

### SAA3 Is Expressed by Colonic Epithelial Cells and Intraepithelial Macrophages

**Figure 5:**
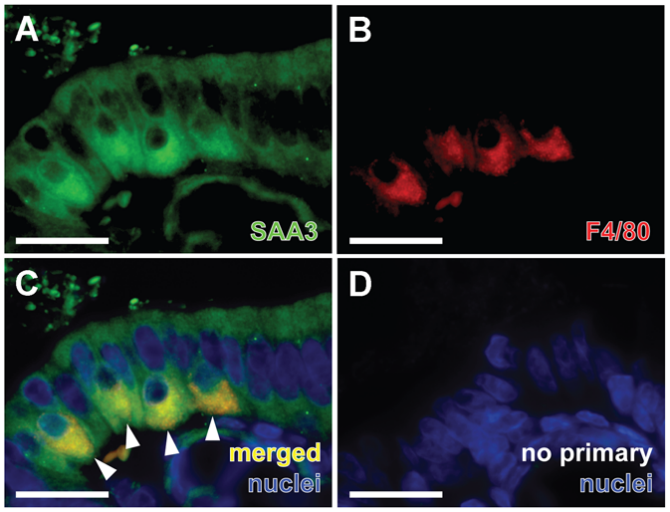
SAA3 is expressed in both colonic epithelial cells and intraepithelial macrophages. (A) Multilabel immunohistochemistry revealed that multiple cells in the CONV-R mouse colonic epithelium express SAA3 (*green*). (B) Presence of F4/80-positive cells (*red*; pan-macrophage marker) by intraepithelial macrophages. (C) Arrowheads point to four intraepithelial macrophages (positive for SAA3 and F4/80) at the luminal interface with bacteria. Nuclei are stained with *bis*-benzimide (*blue*). (D) Representative staining with primary antibodies omitted. Scale bars = 30 µm.

Co-staining colonic sections from CONV-R mice with the pan-macrophage marker F4/80 demonstrated that SAA3 is expressed not only by intestinal epithelial cells but also by intraepithelial macrophages (Figures 5A–5D). Identification of SAA3 expression in both epithelial cells and macrophages in the colonic mucosa suggested that both cell types may contribute to the elevated SAA3 levels identified in the colon in the presence of gut microbiota.

### LPS Induces SAA3 and TNF-α Expression in Colonic Epithelial Cells and Macrophages

**Figure 6:**
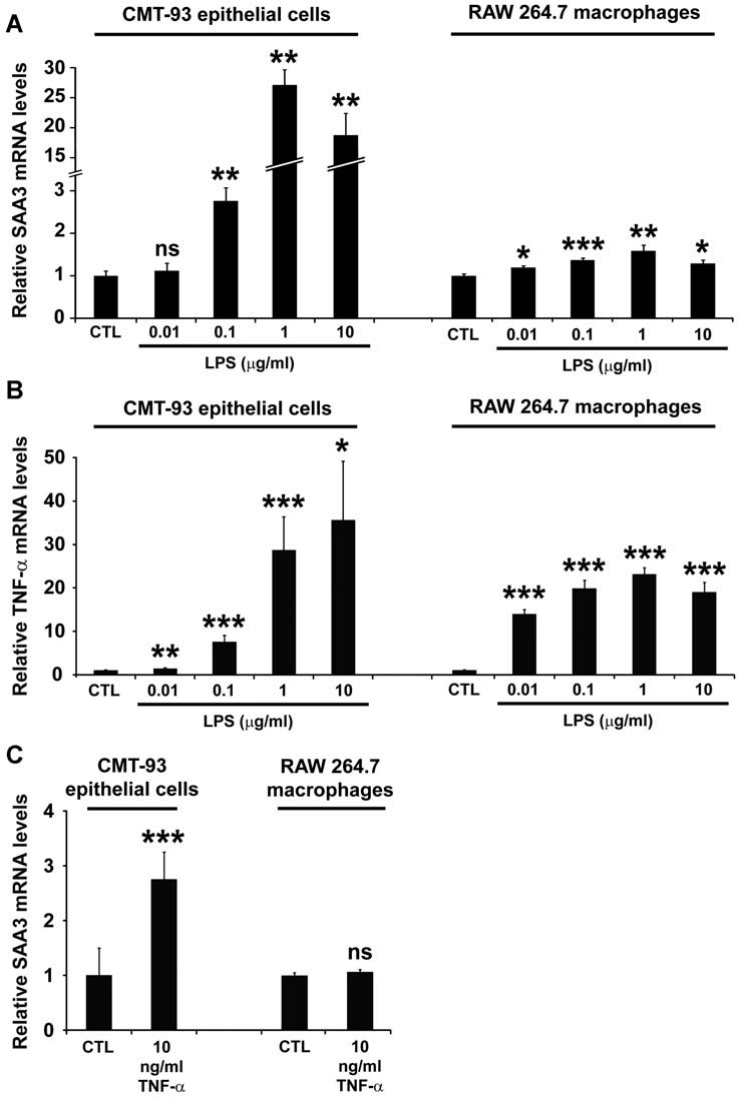
LPS induces SAA3 and TNF-α mRNA expression in CMT-93 colonic epithelial cells and RAW 264.7 macrophages. (A) qRT-PCR analysis of SAA3 mRNA expression levels in CMT-93 cells and RAW 264.7 macrophages after 1 h LPS at the indicated concentrations. (B) qRT-PCR analysis of TNF-α mRNA expression levels in CMT-93 cells and RAW 264.7 macrophages after 1 h LPS at the indicated concentrations. (C) SAA3 mRNA expression levels in CMT-93 cells and RAW 264.7 macrophages after 1 h treatment with recombinant TNF-α (10 ng/ml). n = 6 biological replicates per treatment. *P<0.05, **P<0.01, ***P<0.001; Student's *t*-test.

LPS is an abundant proinflammatory microbial product in the intestinal lumen of mice and humans [33]. To test the relative expression of SAA3 mRNA in both colonic epithelial cells and macrophages stimulated with LPS, we treated the CMT-93 colonic epithelial cell line and RAW 264.7 mouse macrophages with increasing concentrations of purified *E. coli* LPS (Figure 6A). In the CMT-93 cells, LPS induced a concentration-dependent increase in SAA3 mRNA levels, with a 27-fold increase observed at 1 µg/ml. LPS (1 µg/ml) also induced binding of nuclear proteins in these epithelial cells to NF-κB binding sites (Figure S3). Accordingly, LPS treatment increased SAA3 mRNA levels in RAW 264.7 macrophages, but the maximal increase was only 1.6-fold at 1 µg/ml LPS.

As expected, LPS treatments elicited significant increases in TNF-α expression in both CMT-93 cells and RAW 264.7 macrophages (Figure 6B). In both cell types, TNF-α mRNA was dependent on LPS concentration within the range we tested. The most pronounced induction was observed at 10 µg/ml LPS in CMT-93 cells (36-fold) and at 1 µg/ml LPS in macrophages (23-fold).

Recombinant mouse TNF-α protein induced significant SAA3 transcription in CMT-93 cells (2.7-fold) but not in RAW 264.7 macrophages (Figure 6C).

## Discussion

We investigated the effect of gut microbiota on extrahepatic SAA3 levels by comparing samples from GF and CONV-R mice, and showed that expression of SAA3 in adipose tissue and colonic tissue was augmented in the presence of gut microbiota. TNF-α expression was also increased in colonic tissue from CONV-R mice. We provided evidence suggesting that the microbiota-induced increase in colonic SAA3 expression may be partially mediated through the TLR/MyD88/NF-κB signaling pathway. Furthermore, SAA3 was expressed both by the mouse colonic epithelium and intraepithelial macrophages *in vivo*. In cell culture experiments, LPS induced a greater increase in baseline-relative SAA3 expression in colonic epithelial cells than in macrophages. In contrast, LPS induced similar fold-change differences in TNF-α expression in both cell types.

We showed a significant upregulation of SAA3 but not SAA1/2 in adipose tissue from CONV-R compared with GF mice. Comparison of the amino acid sequence alignments of SAA isoforms in mice and humans demonstrated that mouse SAA3 is most similar to the human isoform SAA1 (70% amino acid identity; Table S1). SAA1 expression is increased in hypertrophic adipocytes from obese humans [34] and has been shown to augment lipolysis in adipocytes [19]. Thus we speculated that mouse SAA3, which is upregulated in the adipose tissue of obese mice [18], might be functionally similar to human SAA1 and perhaps represent a link between low-grade inflammation and obesity.

The increased SAA3 expression in adipose tissue from CONV-R mice could be: (1) a direct response to systemic LPS originating from the gut microbiota, (2) due to increased levels of pro-inflammatory cytokines that promote adipose SAA3 expression, or (3) an indirect consequence of increased adipocyte size, which has been shown for SAA1 in hypertrophic human adipocytes [34]. We have identified increased LPS levels in the serum of CONV-R compared to GF mice (C. S. R., G. Ö. L. and F. B., unpublished observation) and it is known that SAA3 expression in adipose tissue *in vivo* is induced 18 h after LPS injection [26]. However, we cannot rule out an indirect effect of the gut microbiota as epididymal fat pads and adipocytes from CONV-R mice are larger than those from GF mice [8].

SAA3 expression in the mouse colon was primarily localized to the colonic epithelium. To examine whether microbiota-induced increases in colonic SAA3 expression could potentially be mediated through the TLR4/MyD88/NF-κB signaling pathway, we first established that TLR4 was present in the colonic epithelium. We then showed that SAA3 expression was significantly reduced in *Myd88−/−* mice. Furthermore, we detected NF-κB binding in colonic nuclear extracts from all but one of the CONV-R mice we assessed, but never in any extract from a GF mouse. This variation in NF-κB responses in CONV-R mice is intriguing and consistent with the increased biological variation in colonic SAA3 expression of CONV-R mice (*e.g.*, the relative SAA3 levels calculated in Figure 2B within the CONV-R group had a larger standard error of the mean compared with GF counterparts). Consistent with previous studies that components of the LPS/CD14/TLR4 signaling cascade is required for metabolic inflammation and development of obesity [14], [15], we observed that, compared with WT mice, *Myd88-*deficient mice gained significantly less weight on the high-fat Western diet and possessed significantly lower epididymal fat pad and liver masses relative to total body weight (C. S. R. and F. B., unpublished data).

We propose that LPS and potentially other products of gut bacteria might activate TLR receptors and mediate signaling through MyD88 and NF-κB to promote increased SAA3 expression. However, we cannot exclude the involvement of additional signaling pathways also leading to NF-κB activation, nor can we exclude the possibility that MyD88-independent induction of colonic SAA3 expression could be due to downstream pathways other than those involving NF-κB (*i.e.*, MAPK and PI3K pathways). Furthermore, additional experiments involving chromatin immunoprecipitation or EMSAs targeting known NF-κB binding sites in the promoters regions of SAA3, TNF-α and other cytokines would be useful to verify our proposed mechanism and assess the breadth of its impact in different tissues.

Activation of SAA3 through TLR4 would be intriguing in light of a recent study demonstrating that SAA3 can itself act as an agonist of TLR4 and activates NF-κB in pre-metastatic mouse lung macrophages and endothelial cells [35]. In principle, a feed-forward loop involving TLR4 and SAA3 could exacerbate chronic inflammatory diseases under certain circumstances. We initially speculated that SAA3 could influence inflammation peripherally by means of its secretion into the bloodstream. However, mass spectrometric analysis of serum from CONV-R mice only identified SAA1, SAA2 and SAA4 in the peripheral circulation (data not shown) which is consistent with a recent publication [25]. Thus colonic SAA3 may have predominantly autocrine and/or paracrine functions and promote inflammation by both activating TLR4 [35] and acting as monocyte chemoattractant [36].

We detected SAA3 expression in both colonic epithelial cells and macrophages, and continued our studies using cell lines to investigate the relative induction levels in each cell type. Although the magnitude of LPS-mediated stimulation of TNF-α expression was similar in both CMT-93 colon epithelial cells and RAW 264.7 macrophages, the relative increase of SAA3 mRNA expression in response to LPS was considerably greater in the CMT-93 cells. Previous studies have shown that TNF-α induces SAA3 in mouse adipocyte and granulosa cell culture models [17], [27], and here we showed that recombinant TNF-α induced SAA3 expression in CMT-93 cells but not in the macrophage cell line. Thus, although the increased TNF-α expression levels in colonic tissue from CONV-R mice could be derived from both colonic epithelial cells and macrophages, our data suggest that TNF-α at 10 ng/ml will only induce SAA3 expression in epithelial cells, whereas SAA3 expression in macrophages may be constitutive. There are many more epithelial cells than macrophages in the colon, and neither the microbiota nor *Myd88* genotype has been shown to have a significant effect on the number of macrophages present in colonic villi [37]. Thus, microbially augmented SAA3 levels in the mouse colon may be derived primarily from epithelial cells rather than macrophages. However, as the basal levels of SAA3 protein expression by individual colonic macrophages appear higher than those of individual epithelial cells (Figure 5), even a small increase in relative expression could have a significant biological impact and perhaps correspond to greater absolute expression levels *in vivo*, especially in regions of high macrophage density.

**Figure 7:**
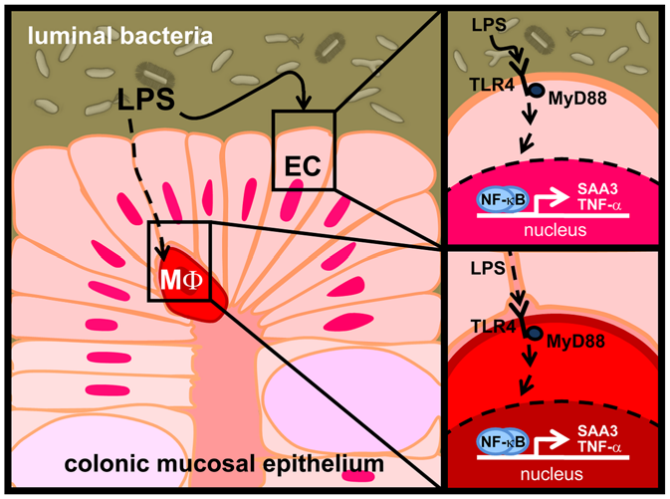
A model for microbial induction of SAA expression in the mouse colon. SAA3 and TNF-α activation in the colon may occur upon (*left panel*) direct stimulation of epithelial cells by LPS or translocation of LPS across compromised tight junctions. (*upper right panel*) Colonic epithelial cells (EC) express TLR4 and respond to LPS stimulation by importing NF-κB to the nucleus where it can bind to DNA regulatory elements and promote expression of SAA3. LPS also stimulates the expression of TNF-α, another cytokine influencing local and systemic inflammation. TNF-α may further stimulate SAA3 expression in the intestinal epithelium. (*lower right panel*) Macrophages (MΦ) also express TLR4 and respond to LPS stimulation by promoting transcription of TNF-α mRNAs.

In summary, we showed that SAA3 expression in the mouse colon is increased by the presence of the gut microbiota, and that this may be partially mediated through the TLR/MyD88/NF-κB signaling pathway. We demonstrated that LPS is a potent inducer of SAA3 expression in cultured colonic epithelial cells and suggest a model (Figure 7) whereby the gut microbiota significantly induces both SAA3 and TNF-α expression in colonic epithelial cells and macrophages; however, based on our cell culture models, the fold-induction of TNF-α in macrophages may be higher than is observed for SAA3. Although the specific roles of SAA3 in colonic and adipose tissue are not well understood, in consideration of recent studies, it is possible that elevation of SAA3 in mice (and potentially SAA1 in humans) by TLR/MyD88/NF-κB signaling downstream of LPS or other components of the intestinal microbiota represents a facet of the elevated inflammatory tone observed in obesity. We speculate that in the future, manipulation of molecular interactions between specific proinflammatory microbial products and responsive host cells could develop into therapeutic approaches to reduce low-grade chronic inflammation and help to alleviate associated metabolic complications that influence insulin resistance and obesity.

## Materials and Methods

### Mouse Studies

10- to 12-week-old GF Swiss Webster male mice were maintained in flexible film isolators under a strict 12-h light cycle. GF status was verified regularly by ensuring negative cultures from mouse feces in three media types: Nutrient Broth (Merck), Brain Heart Broth (Merck), and Sabouraud Liquid Medium (Oxoid) and monitoring by PCR for bacterial 16S rDNA. Conventionally raised (CONV-R) Swiss Webster mice were transferred into identical isolators at weaning. Both groups of mice were fed an autoclaved chow diet (Labdiet) *ad libitum*. *Myd88*−/− mice and wild type (WT) C57Bl/6J controls were maintained in microisolator cages on a standard chow diet for 8–10 weeks and then switched to a high-fat, high-sugar “Western” diet (Adjusted Calorie Diet, 42% fat; Harlan-Teklad) for 8 weeks. All manipulations involving mice were performed using protocols approved by the local animal ethics committee at the University of Gothenburg.

Mice were killed after a 4 h fast. White adipose tissue was harvested from the epididymal, mesenteric and perinephric regions. Small intestines were resected and divided into eight equal segments. The first, fourth, and eighth segments were designated duodenum, jejunum and ileum, respectively. Analyses of colonic tissues were performed on the proximal 2 cm of the large intestine, adjacent to the cecum. Tissues harvested for RNA or protein analyses were frozen immediately in liquid nitrogen and stored at −80°C until further processed.

### Cell Culture

Both the mouse colonic epithelial cell line (CMT-93) and the macrophage cell line (RAW 264.7) were purchased from ATCC (Manassas, VA) and maintained in Dulbecco's Modified Eagle Medium (PAA Laboratories) containing 100 U/mL penicillin, 100 µg/ml streptomycin, 2 mM *L*-glutamine, 2 mM sodium pyruvate and 10% fetal calf serum. Cells were seeded at equal densities in 6-well plates and, after reaching confluence, they were washed and incubated for one additional hour in the same medium (control) or medium supplemented with γ-irradiated LPS purified from *E. coli* serotype 055∶B5 (Sigma-Aldrich) or mouse recombinant tumor necrosis factor-α (TNF-α, 410-MT; R&D Systems). Cells in individual wells were then analyzed either by quantitative real-time PCR or in electrophoretic mobility shift assays (EMSAs).

### Quantitative Real-Time PCR

Cultured cells for RNA analyses were homogenized by passage 5–10 times through a 20-gauge needle fitted to an RNase-free syringe. Tissues for RNA analyses were homogenized using a TissueLyzer (Qiagen). All homogenates were processed for RNA isolation using an RNeasy kit (Qiagen) with on-column DNase I (Qiagen) treatment according to the manufacturer's instructions. Random hexamer-primed cDNA templates were synthesized from purified RNAs using the High Capacity cDNA Reverse Transcription Kit (Applied Biosystems) according to the manufacturer's instructions. qRT-PCR assays were performed in 25-µl reactions containing 1× SYBR Green Master Mix buffer (Thermo Scientific), and 900 nM gene-specific primers (300 nM primer concentrations were used to assess L32 transcripts). A melting curve was performed for each primer pair to identify a temperature where only amplicon, and not primer dimers, accounted for SYBR Green-bound fluorescence. Assays were performed in analytical duplicates or triplicates using a 7900HT Fast Real-Time PCR System (Applied Biosystems) or CFX96 Real-Time System (Bio-Rad Laboratories) and normalized to the level of RNA encoding the L32 ribosomal protein using the ΔΔC_T_ analysis method [38]. SAA1 and SAA2 (SAA1/2) were analyzed as one transcript because of the high sequence homology. Primer sequences used in this study can be found in Table S2.

### Multilabel Immunohistochemistry

Tissues to be used for immunohistochemical staining were resected and immediately fixed in 4% zinc formaldehyde prior to embedding in paraffin blocks. Paraffin-embedded tissues were sectioned to a thickness of 5 µm and affixed to glass slides. Slides were deparaffinized and processed for antigen retrieval with a 2100 Retreiver using 1× DIVA solution and Hot Rinse (HistoLab Products AB). Sections were rinsed with deionized water for 5 min and incubated for 1 h at room temperature in blocking buffer (1× PBS with 0.05% Tween (PBS-T) and 1% bovine serum albumin (Sigma-Aldrich)). Rabbit anti-mouse polyclonal SAA3 antibody [17] (a generous gift from Philipp Scherer) was diluted 1∶200 in blocking buffer either alone or with a rat anti-F4/80 primary antibody (pan-macrophage marker; 1∶600 in blocking buffer; Abcam) and incubated overnight at 4°C. Rabbit polyclonal antibody to TLR4 (AbCam) was used at 1∶50 in blocking buffer to detect TLR4 in mouse colon. Following three washes in PBS-T, antigen-antibody complexes were visualized with Alexa Fluor 488- or 594-conjugated secondary antibodies (1∶2000 in blocking buffer for 1 h; Molecular Probes). Nuclei were stained with *bis*-benzimide (1 µg/ml; Sigma-Aldrich) in the second of three washes with PBS-T and mounted in a 1∶1 mixture of PBS∶glycerol. Stained sections were imaged with an Axioplan 2 microscope equipped with an AxioCam HRc camera (Zeiss).

### Electromobility Shift Assays

Nuclear proteins were isolated from either confluent CMT-93 cells in culture or 100 mg of proximal colon tissue from GF and CONV-R mice (n = 6 mice per group) using the CelLytic NuCLEAR Extraction Kit (Sigma-Aldrich). Nuclear extracts were mixed with reagents from the LightShift Chemiluminescent EMSA Kit (Pierce Biotechnology) at 4°C for 20 min and then again at 4°C for 20 min after addition of double-stranded, biotinylated probes containing an NF-κB binding site [39]. Sequences of the annealed oligonucleotides can be found in Table S2. Additional probes for competition experiments (one with the same NF-κB binding site and another with a mutated NF-κB binding site to ensure specificity) were unlabeled and used in 200-fold excess. DNA-protein complexes were separated on a 6% DNA Retardation Gel (Invitrogen) at 100 V for 1 h, transferred to nitrocellulose membrane, exposed to chemiluminescence film and visualized according to manufacturers' instructions.

### Coding Sequence Alignment

Comparisons of mouse and human SAA coding sequences were performed by nucleotide and amino acid alignment using BLAST 2 sequences [40]. BLAST parameters and the accession numbers corresponding to analyzed sequences can be found with coding sequence alignment results in Table S1.

### Statistical Analysis

Unless otherwise stated, differences between experimental groups were based on assessment of individual mice (n = 7−13 per group) or cell culture treatments (n = 6 independent biological replicates) and reported as mean fold difference±SEM. Statistical significance was assessed by Student's two-tailed *t*-test. Data were considered significant when *P*<0.05. Assessment of relative SAA3 mRNA expression along segments of the mouse small intestine and colon was performed using pooled cDNA from 5 mice per group in analytical triplicates with results reported as mean fold difference±SD.

## Supporting Information

Figure S1 (8.06 MB PDF)

Figure S2 (1.37 MB PDF)

Figure S3 (8.06 MB PDF)

Table S1 (0.01 MB PDF)

Table S2 (0.05 MB PDF)
