# Supplementary material for: Regulation of Serum Amyloid A3 (SAA3) in Mouse Colonic Epithelium and Adipose Tissue by the Intestinal Microbiota
Source: PLoS One. 2009 Jun 9;4(6):e5842. doi: 10.1371/journal.pone.0005842 (PMC2688757; doi:10.1371/journal.pone.0005842)
Supplement: Figure S1 — (8.06 MB PDF) [file pone.0005842.s001.pdf]

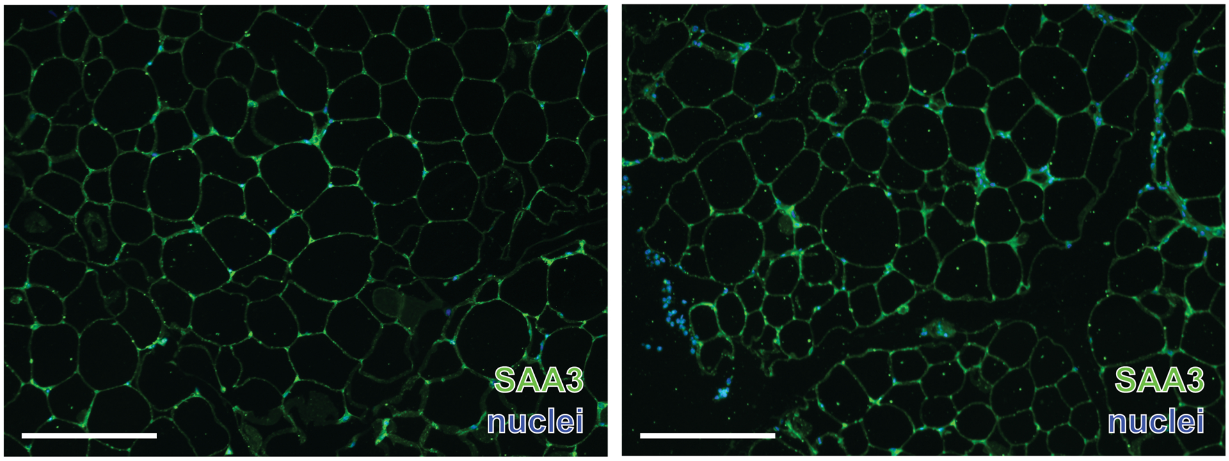

perinephric adipose tissue **mesenteric adipose tissue**

Figure S1. Representative immunostaining of perinephric and mesenteric white adipose tissue for SAA3 (*green*) with nuclei (*blue*) labeled with *bis*-benzimide. *Bars* = 200 μm.
