# Supplementary material for: Regulation of Serum Amyloid A3 (SAA3) in Mouse Colonic Epithelium and Adipose Tissue by the Intestinal Microbiota
Source: PLoS One. 2009 Jun 9;4(6):e5842. doi: 10.1371/journal.pone.0005842 (PMC2688757; doi:10.1371/journal.pone.0005842)
Supplement: Figure S2 — (1.37 MB PDF) [file pone.0005842.s002.pdf]

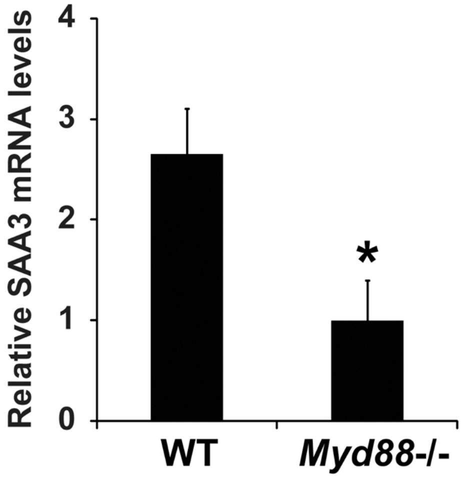

Figure S2. SAA3 mRNA expression in white adipose tissue is significantly lower in *Myd88*-/- mice compared with that of wild type C57Bl/6 controls (n = 10 mice per group). *P < .05; Student's t test. Both groups were raised with a conventional microbiota.
