# Supplementary material for: Regulation of Serum Amyloid A3 (SAA3) in Mouse Colonic Epithelium and Adipose Tissue by the Intestinal Microbiota
Source: PLoS One. 2009 Jun 9;4(6):e5842. doi: 10.1371/journal.pone.0005842 (PMC2688757; doi:10.1371/journal.pone.0005842)
Supplement: Figure S3 — (8.06 MB PDF) [file pone.0005842.s003.pdf]

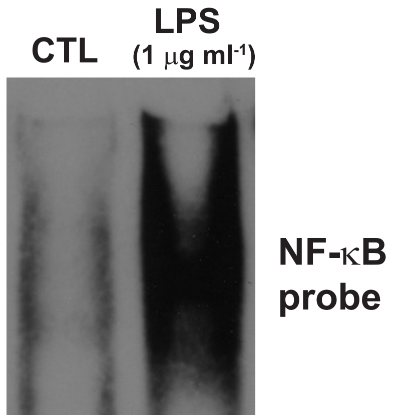

Figure S3. EMSA revealed that nuclear extracts from CMT-93 colonic epithelial cells treated with LPS bind to a biotinylated NF-κB probe.
