# Supplementary material for: Regulation of Serum Amyloid A3 (SAA3) in Mouse Colonic Epithelium and Adipose Tissue by the Intestinal Microbiota
Source: PLoS One. 2009 Jun 9;4(6):e5842. doi: 10.1371/journal.pone.0005842 (PMC2688757; doi:10.1371/journal.pone.0005842)
Supplement: Table S1 — (0.01 MB PDF) [file pone.0005842.s004.pdf]

Table S1. Mouse and Human SAA Coding Sequence Alignments

MOUSE (mSAA) AND HUMAN (hSAA) CODING SEQUENCES

	CCDS	NCBI protein ID	Protein (aa)
mSAA1	CCDS 21284.1	NP_033143.1	122
mSAA2	CCDS 21285.1	NP_035444.1	122
mSAA3	CCDS 21282.1	NP_035445.1	122
mSAA4	CCDS 21283.1	NP_035446.1	130
hSAA1*	CCDS 7835.1	NP_000322.2<br>NP_954630.1	122
hSAA2	CCDS 7833.1	NP_110381.2	122
hSAA3**	n/a	AAO48437.1	60
hSAA4	CCDS 7832.1	NP_006503.1	130

*transcript variants identified (same predicted CDS)
**no consensus transcript in CCDS database, comparisons use product of an identified sequence variant

BLAST ALIGNMENT REGIONS: MOUSE SAA ISOFORMS AGAINST HUMAN SAA ISOFORMS

	hSAA1	hSAA2	hSAA3	hSAA4
mSAA1	88/122	85/122	27/48	68/130
mSAA2	88/122	85/122	26/48	68/130
mSAA3	86/122	74/96	31/48	67/130
mSAA4	71/130	56/104	27/52	75/130

PERCENT IDENTITY ACROSS ENTIRE CODING SEQUENCE***

	hSAA1	hSAA2	hSAA3	hSAA4
mSAA1	72	70	22	52
mSAA2	72	70	21	52
mSAA3	70	61	25	52
mSAA4	55	43	21	58

Default BLASTP settings:
Matrix: BLOSUM62; gap open: 11; gap extension: 1; x_dropoff: 0; expect 10; wordsize: 3

*** Percent identity is calculated as: $\frac{100 \times \text{(the number of amino acids that align using default BLAST parameters)}}{\text{(the total number of amino acids in the longest isoform in that comparison)}}$
