# Supplementary material for: Regulation of Serum Amyloid A3 (SAA3) in Mouse Colonic Epithelium and Adipose Tissue by the Intestinal Microbiota
Source: PLoS One. 2009 Jun 9;4(6):e5842. doi: 10.1371/journal.pone.0005842 (PMC2688757; doi:10.1371/journal.pone.0005842)
Supplement: Table S2 — (0.05 MB PDF) [file pone.0005842.s005.pdf]

Table S2. Oligonucleotide Sequences Used in this Study

**Bacterial Screening:**	**DNA Sequence (5'-3')**
Universal27 forward	AGAGTTTGATCCTGGCTCAG
Universal1391 reverse	GACGGGCGGTGWGTRCA
***Myd88* Genotyping:**	
Myd88 forward	TGGCATGCCTCCATCATAGTTA
Myd88 reverse	GTCAGAAACAACCACCACCAT
Myd88-lac reverse	ATCGCCTTCTATCGCCTTCTTG
**Quantitative Real-Time PCR:**	
L32 forward	CCTCTGGTGAAGCCCAAGATC
L32 reverse	TCTGGGTTTCCGCCAGTTT
SAA1/2 forward	GTAATTGGGGTCTTTGCC
SAA1/2 reverse	TTCTGCTCCCTGCTCCTG
SAA3 forward	TGCCATCATTCTTTGCATCTTGA
SAA3 reverse	CCGTGAACTTCTGAACAGCCT
TNF-α forward	CCAGACCCTCACACTCA
TNF-α reverse	CACTTGGTGGTTTGCTACGAC
TLR4 forward	ATGGCATGGCTTACACCACC
TLR4 reverse	GAGGCCAATTTTGTCTCCACA
**Electromobility Shift Assays:**	
NF-κB biotinylated +	biotin-AGTTGAGGGGACTTTCCCAGGC
NF-κB biotinylated -	biotin-GCCTGGGAAAGTCCCCTCAACT
NF-κB unlabeled +	AGTTGAGGGGACTTTCCCAGGC
NF-κB unlabeled -	GCCTGGGAAAGTCCCCTCAACT
NF-κB mutated +	AGTTGAGCTCACTTTCCCAGGC
NF-κB mutated -	GCCTGGGAAAGTGAGCTCAACT
